# Integrated Bioinformatics Analysis Revealing that the *NSDHL* Gene Might Be Associated with the Progression of Western HFD/SW-Induced Hepatocellular Carcinoma

**DOI:** 10.2174/0118715303356256250129063049

**Published:** 2025-02-28

**Authors:** Jing Hong, Bo Pan, Zhaoxian Yan, Xiao-feng Zhai, Yongshang Liu

**Affiliations:** 1 Department of Integration of Chinese and Western Medicine, Peking University Cancer Hospital & Institute, Beijing, 100142, China;; 2 Department of Integrative Oncology, The First Affiliated Hospital of Naval Medical University, Shanghai 200433, China;; 3 School of Traditional Chinese Medicine, Naval Medical University, Shanghai 200433, China;; 4 School of Integrative Medicine, Shanghai University of Traditional Chinese Medicine, Shanghai 201203, China;; 5 Department of Spleen and Stomach, Baiyun Hospital of The First Affiliated Hospital of Guangzhou University of Chinese Medicine, Guangzhou 510470, Guangdong Province, China

**Keywords:** Bioinformatics analysis, diet, hepatocellular carcinoma, *NSDHL*, hub genes, biomarker

## Abstract

**Background and Objective:**

Hepatocellular carcinoma (HCC) remains a significant global health concern. However, the etiology and pathogenesis of HCC have yet to be fully elucidated. Previous studies have indicated a close association between obesity and the occurrence and progression of HCC. The objective of this study was to employ bioinformatics strategies in order to explore key genes associated with the clinical diagnosis and prognosis of HCC induced by a Western high-fat diet and sugar water (HFD/SW).

**Materials and Methods:**

We obtained the expression profile chip data GSE197884 from the Gene Expression Omnibus (GEO) database. Subsequently, “DESeq” and “Limma” R packages were employed to identify differentially expressed genes (DEGs) while constructing a co-expressed gene network using weighted gene co-expression analysis (WGCNA). Functional enrichment analyses were then carried out, followed by the construction of a protein-protein interaction (PPI) network to uncover core genes. The core genes were confirmed through data retrieved from The Cancer Genome Atlas (TCGA) database in order to determine their status as hub genes. Finally, survival and tumor immune infiltration analyses were performed to unveil the prognostic significance of these hub genes.

**Results:**

In total, 126 intersection targets were retrieved through the Venn diagram. Gene ontology (GO) enrichment and Kyoto Encyclopedia of Genes and Genomes (KEGG) pathway analyses revealed that the DEGs were primarily related to the proliferation and apoptosis of HCC cells, the digestion and metabolism of liver cells, the HCC tumor microenvironment, and immune response. The PPI network analysis identified 11 core targets, among which seven hub genes, including *NSDHL, MVK, SQLW, GCAT, ALAS2, GLDC*, and *AGXT*, were obtained after TCGA database validation. Furthermore, it was found that *NSDHL* was closely associated with the clinical diagnosis and prognosis of HCC induced by HFD/SW and also affected the cellular immune infiltration in the HCC tumor microenvironment.

**Conclusion:**

The present study demonstrated a significantly elevated expression of *NSDHL* in HCC tissues, suggesting its potential as a specific biomarker for precise clinical diagnosis and prognosis assessment of HCC induced by HFD/SW.

## 1 INTRODUCTION

According to the 2022 *GLOBOCAN* data released by the International Agency for Research on Cancer under the World Health Organization, primary liver cancer ranked sixth in terms of incidence (4.3%) and third in terms of mortality (7.8%) globally [1]. Primary liver cancer encompasses hepatocellular carcinoma (HCC), which constitutes 75%-85% of cases, intrahepatic cholangiocarcinoma accounting for 10%-15%, as well as other rare tumors, such as fibrolamellar carcinoma and hepatoblastoma [2, 3]. The main risk factors for hepatocellular carcinoma (HCC) have been previously identified as chronic hepatitis virus infection, exposure to aflatoxin and aristolochic acid, excessive alcohol consumption, obesity, fatty liver disease, liver cirrhosis, type 2 diabetes mellitus, and smoking [4, 5].

In recent years, the correlation between diet and the incidence of HCC has become a research hotspot [6-8]. Previous studies reported that metabolic dysregulation plays a critical role in HCC development [9-11]. The escalating prevalence of non-alcoholic fatty liver disease (NAFLD) and non-alcoholic steatohepatitis (NASH) has established metabolic disorders as a prominent risk factor for hepatocellular carcinoma (HCC) development [12-14]. The global prevalence of NAFLD is approximately 25%, and the occurrence of NAFLD-associated HCC tends to be more common in the elderly population without cirrhosis, which distinguishes it from liver diseases of other etiologies as it often gets diagnosed at a later stage [15]. Aerobic glycolysis plays a pivotal role in the proliferation, growth, invasion, and therapeutic response of HCC [16]. Otto Warburg discovered that even in the presence of abundant oxygen, a majority of cancer cells exhibit a preference for converting glucose into lactic acid. This metabolic phenomenon, known as aerobic glycolysis or the Warburg effect, is characterized by augmented glucose uptake and subsequent lactic acid production, thereby highlighting significant metabolic reprogramming within the tumor microenvironment. Despite this metabolic pathway resulting in reduced ATP production, it facilitates pyruvate conversion into a precursor for nucleotide synthesis, thereby providing a crucial substrate for cancer cell proliferation [17-19]. Specifically, NAFLD has been considered a key link between obesity and HCC [20-22]. The epidemic of obesity, along with the concomitant development of NALFD, observed in over 85% of obese individuals, is believed to significantly contribute to HCC pathogenesis [23]. A study examining the correlation between body mass index (BMI) and various cancers in a cohort of 781,283 Korean men over a 10-year period revealed that BMI ≥ 30 kg/m^2^ was significantly associated with hepatocellular carcinoma (HCC) incidence [24]. Nair *et al.* [25] analyzed 19,271 liver cirrhosis patients undergoing liver transplantation in the US from 1991 to 2000 and found that obesity was a statistically significant independent risk factor for HCC. After performing a meta-analysis of 11 cohort studies, Larsson *et al.* [16] reported that the relative risks of overweight and obese people with liver cancer were 1.17 and 1.89, respectively, compared to normal-weight people [26].

Recently, a plethora of multi-center genomics studies encompassing transcriptomics, proteomics, and high-throughput sequencing have unveiled a cohort of cancer-associated genes, thereby providing invaluable insights into the intricate molecular mechanisms underpinning disease progression [27, 28]. These genes have been applied to generate slicing data stored in public databases [29]. For example, the Gene Expression Omnibus (GEO), supported by the National Center for Biotechnology Information of the National Library of Medicine, serves as a comprehensive database that accommodates both raw and processed data. It encompasses experimental designs pertaining to high-throughput gene expressions and genomics studies, along with detailed written descriptions and methodologies concerning sample attributes [30, 31]. However, considering that the databases are continually being updated, the uploaded data has not yet been fully utilized. Therefore, the data can be re-integrated and analyzed to identify valuable information for clinical use. Currently, bioinformatics strategies are extensively employed for the analysis of gene expression profiles, while data mining is conducted at the molecular level to unravel the underlying molecular mechanisms of disease progression and predict disease-specific biomarkers. These analyses are expected to have a positive clinical significance since the findings will be applied to improve early diagnosis and prognosis [32, 33].

This study aimed to investigate the key genes associated with the clinical diagnosis and prognosis of HCC induced by a Western high-fat diet and sugar water (HFD/SW) using bioinformatics analysis, thereby offering a novel therapeutic approach for managing HCC resulting from metabolic disorders. We downloaded an original microarray dataset (GSE197884) [34] and analyzed the data using integrated bioinformatics strategies. First, the identification of differentially expressed genes (DEGs) was performed using the “DESeq” and “Limma” packages in R software, which were then intersected with the co-expression genes extracted by the Weighted Gene Co-Expression Network Analysis (WGCNA) package. Subsequently, the DEGs were analyzed for Gene Ontology (GO) functional and Kyoto Encyclopedia of Genes and Genomes (KEGG) pathway enrichment using R software. Furthermore, the protein-protein interaction (PPI) network was constructed using STRING software, followed by the identification of core genes. The expressions of core differential genes were then validated using the liver LIHC module retrieved from the TCGA database. Furthermore, we combined survival and immune cell infiltration analyses to evaluate the role of hub genes in the progression of HCC induced by HFD/SW.

## 2 MATERIALS AND METHODS

### 2.1 Selection of HCC Data and Identification of DEGs

The GSE197884 expression profile dataset was downloaded from the GEO database (https://www.ncbi.nlm.nih.gov/). The dataset displayed the gene expressions in muscular tissues extracted from the livers of 12 mice on a normal diet and 18 mice on a Western HFD/SW diet to perform expression profiling through high-throughput sequencing.

“DESeq” and “Limma” packages in R software version 4.2.0 were applied to identify the DEGs, with “adjusted *P* < 0.05 and fold change (FC)>1 or FC<- 1” as the cutoff values. Heatmaps and volcano plots were generated using the “ggplot2” package in R software.

### 2.2 WGCNA-Based Identification of Key Gene Co-Expression Modules, Differential Expression Analysis, and Intersection with WGCNA Differential Modules

The utilization of co-expression networks enables the identification of potential biomarkers and therapeutic targets. In addition to conducting DEG analysis, we utilized the gene expression profiles from GSE197884 and employed the “WGCNA” R package to construct gene co-expression networks. WGCNA was applied to identify modules of highly correlated genes among samples and establish associations with external sample traits. The top 5,000 genes exhibiting the greatest variance differences in the dataset were utilized for constructing a weighted gene co-expression network. Subsequently, a similarity matrix was generated based on Pearson's correlation coefficient between paired genes. Next, soft powers β = 3 and 20 were selected using the pickSoftThreshold function. The soft threshold power value was selected based on the criterion that the scale-free topology scale R^2^ should exceed 0.85, following which it was converted into a topological overlap matrix (TOM). A hierarchical clustering dendrogram of the 1-TOM matrix was then constructed to classify similar gene expressions into different gene co-expression modules. The genes exhibiting similar expression patterns were classified into distinct modules using average linkage hierarchical clustering, and each module was assigned a unique color label. Genes that could not be assigned to any module were placed in a gray module, which implied that the genes were not co-expressed. The correlation between module eigengenes and clinical traits, including “stage,” in the GSE197884 dataset was assessed using Pearson's correlation test, with a significance level of *P* < 0.05. Notably, gene modules exhibiting the highest correlation coefficient with “stage” were identified as key gene modules associated with HCC progression in mice receiving HFD/SW diet.

The overlapping genes between the DEGs identified by the “Limma” package and the co-expression genes extracted from the co-expression network were utilized to identify potential prognostic genes. These potential prognostic genes were visually represented as a Venn diagram using the “VennDiagram” R package.

### 2.3 Functional and Pathway Analyses of DEGs

The GO, encompassing biological processes (BP), cellular components (CC), and molecular functions (MF) terms, was employed for functional analysis of the DEGs. In addition, the KEGG pathway analysis was conducted to identify the pathways associated with the genes. The “clusterProfiler” R package was applied to conduct both GO and KEGG analyses of DEGs in the livers of mice receiving the HFD/SW diet, with “adjusted *P* < 0.05” as the cutoff.

### 2.4 PPI Network of Intersecting Differential Genes and Screening of Core Genes

The Search Tool for the Retrieval of Interacting Genes (STRING, http://string.embl.de/) was utilized to construct a PPI network of the identified DEGs, which was visualized using Cytoscape software (Version 3.9.1). Subsequently, the Molecular Complex Detection (MCODE) algorithm was employed to screen and identify the most significant modules in the network, with criteria set as MCODE score > 2, node score cutoff = 0.2, and cutoff degree = 2.

### 2.5 Validation of Hub Genes Using TCGA Database

To validate the identified hub genes, we obtained Liver Hepatocellular Carcinoma (LIHC) data (438 samples) from the TCGA database (https://portal.gdc.cancer.gov/). The “DESeq” package in R software 4.2.0 was used to compare the gene expression between HCC patients and healthy controls. Statistical significance was determined at *P* < 0.05 level and fold change (FC)>1 or FC<- 1.

### 2.6 Survival Analysis of Core Genes After Validation with TCGA Database

The GEPIA database (http://gepia.cancer-pku.cn/index.html) was employed to investigate the prognostic significance, including overall survival, first progression, and post-progression survival, of the hub genes validated by the TCGA database. Moreover, the hazard ratio with 95% confidence intervals and the log-rank *p*-value were estimated. *P* < 0.05 was considered statistically significant.

### 2.7 Identification of Immune Cells Associated with Survival Prognosis

The “Immune-Association” module of the TIMER2.0 database (http://timer.comp-genomics.org/) was employed to analyze the immune infiltration expressions of survival prognosis genes in HCC induced by an HFD, followed by elucidating the immunological process through which they affect tumor progression. We also assessed the immune infiltrating cells, encompassing lymphocytes, macrophages, NK cells, and neutrophils, which hold significant prognostic value for patients' outcomes. Additionally, we investigated the potential association between NAD(P)H steroid dehydrogenase-like protein (NSDHL) gene expression in LIHC and levels of immune infiltration.

## 3 RESULTS

### 3.1 Differentially Expressed Genes in Mice Receiving HFD/SW

**Fig. (1):**
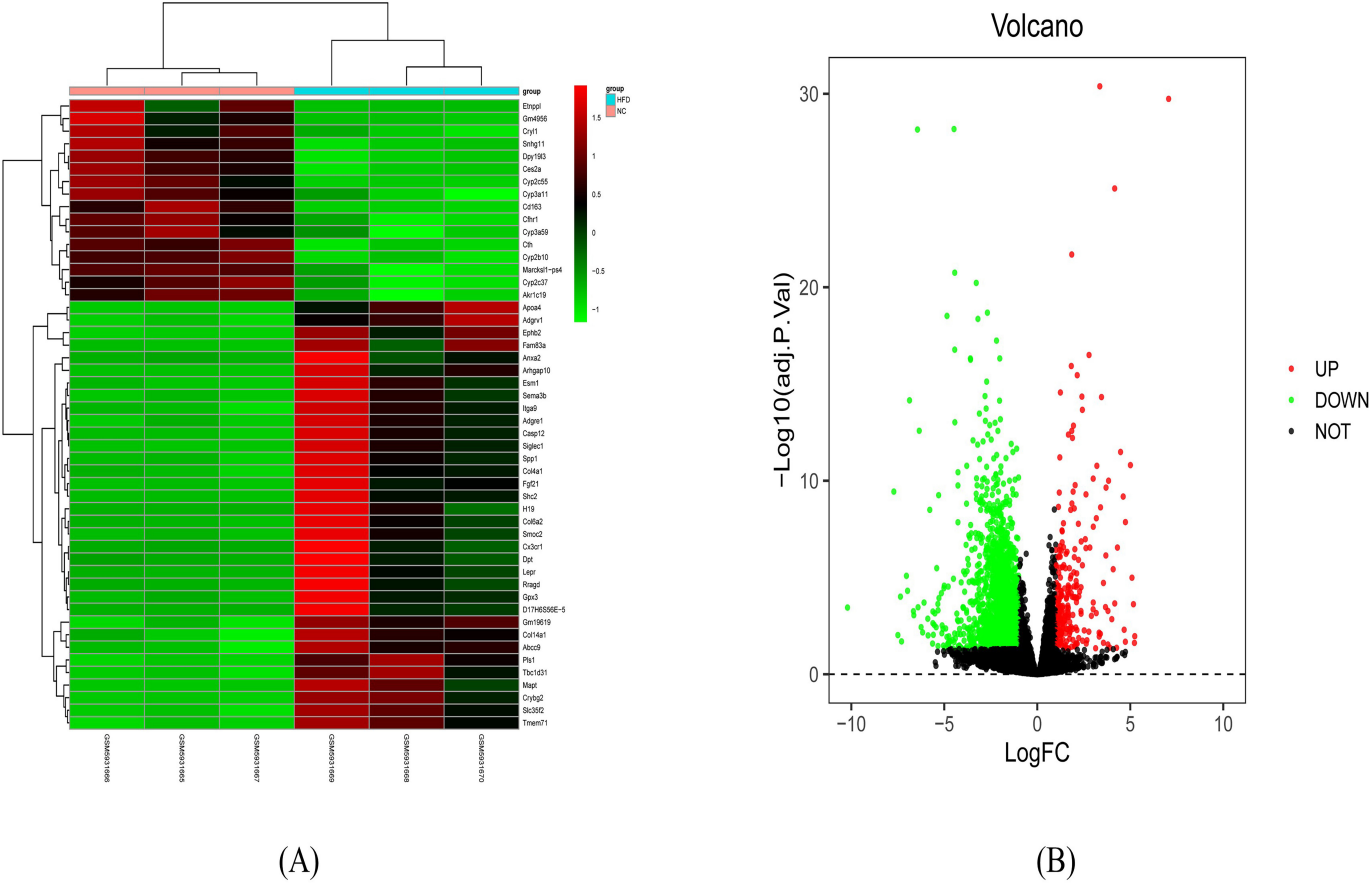
Identification of differentially expressed genes (DEGs) in the GSE197884 datasets of hepatocellular carcinoma (HCC) induced by a Western high-fat diet and sugar water (HFD/SW). (**A**) Heat map of DECs between the HFD/SW and health controls. Colors from green to red mean increasing expression of DECs between the HFD/SW and health controls. (**B**) Identification of DEGs between HFD/SW and health control samples.

Differential gene analysis showed that mice receiving Western HFD/SW had a total of 1,770 DEGs compared to mice on a normal diet, as shown by the volcano plot (Fig. **1A**). A heatmap (Fig. **1B**) was generated to show the expressions of the top 50 DEGs.

### 3.2 Construction of Weighted Gene Co-Expression Modules and Identification of Hub Genes

**Fig. (2):**
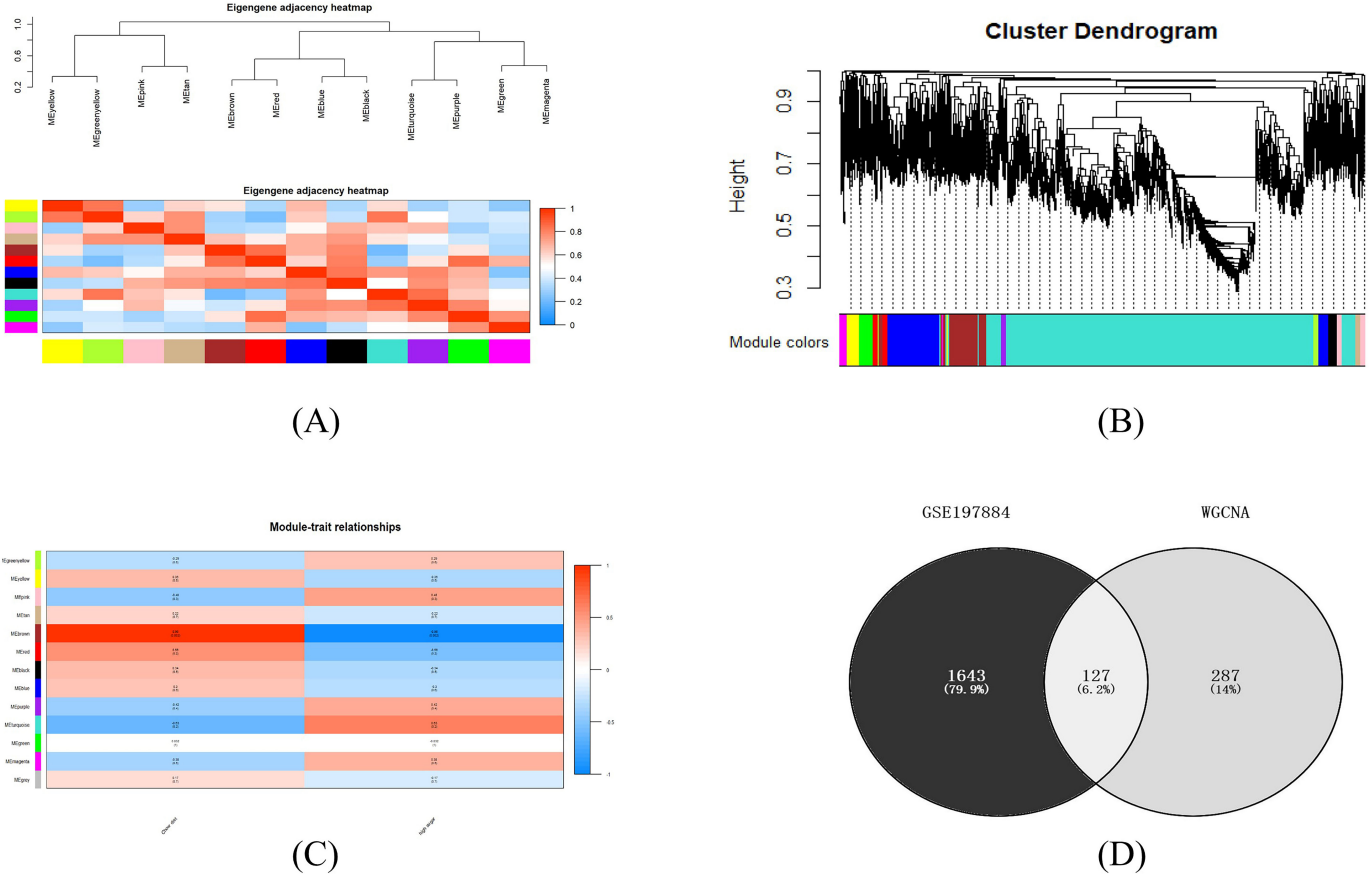
Identification of modules associated with clinical information in the GSE197884 dataset. (**A**) The eigengene adjacency heatmap of GSE197884 was generated. (**B**) The cluster dendrogram of co-expression network modules was hierarchically ordered based on the 1-TOM matrix, representing genes. Each module was assigned a distinct color. (**C**) Module-trait relationships were examined by correlating each color module with clinical traits (HFD/SW and normal). The corresponding correlation coefficient and *P*-value were reported for each cell. (**D**) The Venn diagram illustrates the intersection of DEGs and co-expression modules, revealing a total of 127 overlapping genes.

The “WGCNA” package in R software was used to classify co-expressed DEGs into modules, which were assigned one color. Analysis results showed clustering and dispersion of the different modules of GSE197884 (Fig. **2A**). The DEGs were classified into 12 modules (Fig. **2B**), each represented by a distinct color, except for the gray module, which was unassigned to any cluster. Subsequently, a heatmap illustrating the relationships between modules and two clinical traits (cancer and normal) was generated to assess their associations. The results indicated that the brown module exhibited the highest correlation with normal tissues (brown module: *P*=0.002), including 414 differential genes (Fig. **2C**). Finally, the intersection of the DEG and WGCNA analysis identified a total of 126 target genes (Fig. **2D**).

### 3.3 GO Functional and KEGG Pathway Enrichment Analyses

**Fig. (3):**
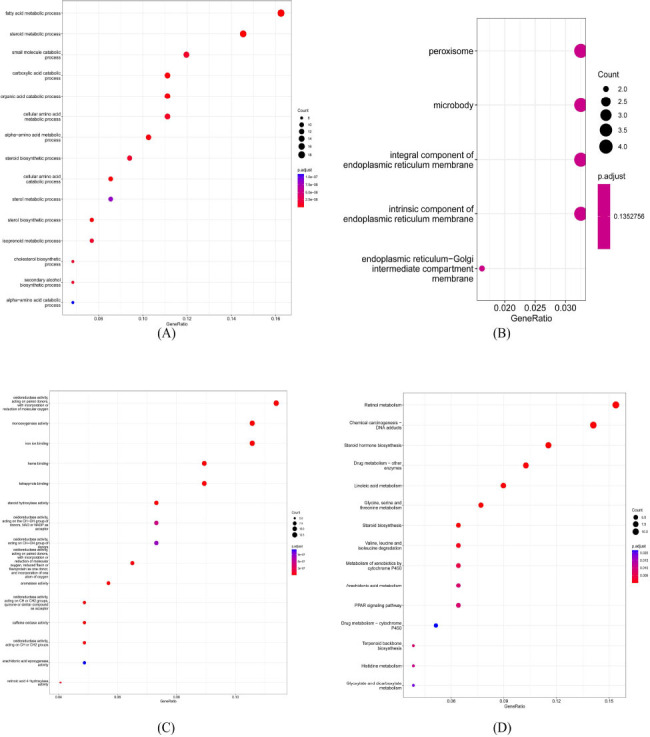
Results of Gene Ontology (GO) and Kyoto Encyclopedia of Genes and Genomes (KEGG) analyses were conducted to investigate the intersecting genes. (**A**) The GO analysis results for cellular components were obtained. (**B**) The GO analysis results for molecular function were obtained. (**C**) The GO analysis results for the biological process were obtained. (**D**) The KEGG pathway results were examined.

The potential functions of the identified intersection genes were investigated through functional enrichment analyses. It was observed that these genes exhibited significant enrichments primarily in fat metabolism, small molecule metabolism, alcohol metabolism, and cellular response (Figs. **3A**-**C**). KEGG pathway analysis demonstrated that the genes were closely associated with retinol metabolism, chemical carcinogens, drug metabolism, amino acid degradation, arachidonic acid metabolism, and PPAR metabolism pathways (Fig. **3D**). Moreover, the results demonstrated that the intersecting genes were implicated in liver cancer cell proliferation and apoptosis, hepatic digestion and metabolism, the tumor microenvironment of liver cancer, as well as immune responses within the body.

### 3.4 Construction of PPI Network and Analysis of Modules

**Fig. (4):**
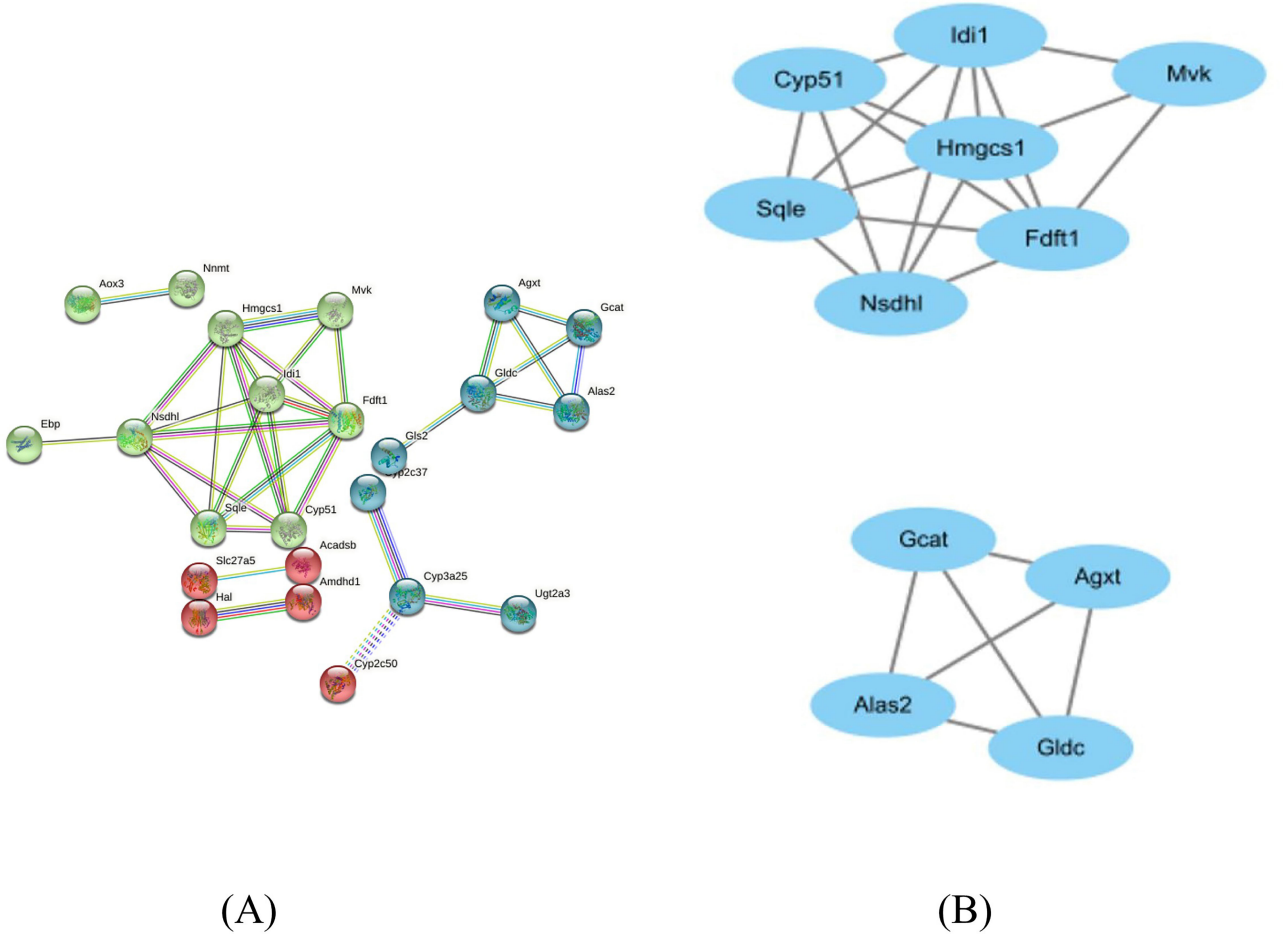
Visualization of the protein-protein interaction (PPI) network and the candidate core genes. (**A**) PPI network of intersecting genes. (**B**) The core gene of intersecting genes.

The PPI network of the intersecting genes was constructed using the STRING database (Fig. **4A**). PPI results were then imported into Cytoscape, and the MCODE plug-in was used to perform core gene analysis. Results identified 11 core DEGs, including *Nsdhl, Cyp51, Mvk, Sqle, Hmgcs1, Fdft1, Gcat, Alas2, Gldc, Agxt*, and *Ldi1* (Fig. **4B**).

### 3.5 Expression Patterns, Prognostic Values, and Protein Expressions of Core DEGs for Validation

The expression levels of the 11 core genes were validated using data from LIHC patients retrieved from the TCGA database. Fig. (**5**) shows that the expression of seven hub genes (*NSDHL, MVK, SQLE, GCAT, ALAS2, GLDC*, and *AGXT*) exhibited significant differences between LIHC tissues and normal tissues. Specifically, *NSDHL* and *SQLE* were upregulated, whereas *MVK, GCAT, ALAS2, GLDC*, and *AGXT* were downregulated.

**Fig. (6):**
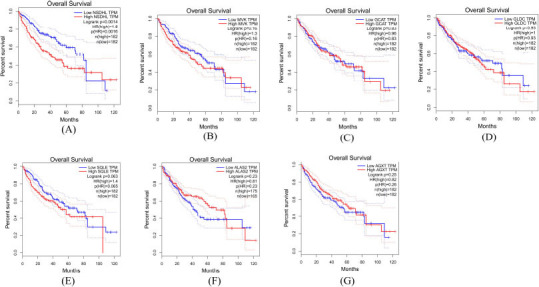
Validation of the expression levels of the seven hub genes was performed in liver hepatocellular carcinoma (LIHC) and normal tissues obtained from the TCGA database. (**A**) Survival analysis was conducted for *NSDHL* in LIHC. (**B**) Survival analysis was performed for MVK in LIHC. (**C**) Survival analysis was carried out for GCAT in LIHC. (**D**) Survival analysis was executed for GLDC in LIHC. (**E**) Survival analysis was undertaken for SQLE in LIHC. (**F**) Survival analysis was implemented for ALAS2 in LIHC. (**G**) Survival analysis was examined for AGXT in LIHC.

In addition, the GEPIA database was employed to perform a Kaplan-Meier analysis of the seven hub genes, with results showing that the expression of *NSDHL* was closely associated with LIHC (Fig. **6**). The expression level of *NSDHL* exhibited a significant correlation with the prognosis of crossed LIHC, highlighting its potential as a prognostic indicator (*P* < 0.0014), suggesting that the gene might be related to the prognosis of LIHC induced by high-fat and high-sugar diets.

### 3.6 Correlation between Immune Infiltration and Expression of *NSDHL* in LIHC

Considering the pivotal role of immune infiltration in tumor genesis and progression, we investigated the impact of *NSDHL* on LIHC progression through the modulation of immune infiltration. Our findings revealed a positive correlation between *NSDHL* expression and LIHC purity (rho=0.145, *P* < 7.00 e-3). Fig. (**7**) shows that the expression of *NSDHL* was positively correlated with B cells (rho=0.213, *P* < 6.81e-5) and neutrophils (rho=0.172, *P* < 1.33e-3) but negatively correlated with CD4+ cells (rho=-0.164, *P* < 0.232e-3) and monocytes (rho=-0.107, *P* < 4.70e-2). These findings suggest that *NSDHL* plays a pivotal role in modulating immune infiltration within the tumor microenvironment of LIHC.

## 4 DISCUSSION

Cancer is a prominent contributor to global mortality and represents the primary impediment to achieving optimal life expectancy for the worldwide population. Primary liver cancer ranks among the top cancers in incidence and mortality, with HCC accounting for the largest proportion of all primary liver cancers [35-37]. HCC is mainly associated with chronic liver diseases, and its incidence depends on the complex interactions among hosts, diseases, and environmental factors [38-40]. Although there are various treatment strategies for HCC, including surgery, radiotherapy, transcatheter arterial chemoembolization, molecular targeted therapy, and immunotherapy, the overall efficacy is not significant [41-43]. Therefore, this calls for studies to identify significant biomarkers, which will aid in understanding the clinical progression and prognosis of HCC. Currently, bioinformatics strategies have been used to explore the diagnostic and prognostic markers affecting HCC. However, the role of metabolic disorders in the development of HCC has attracted widespread attention [41-43]. In this study, we utilized bioinformatic methods to identify potential biomarkers for HCC induced by HFD/SW.

Current high-throughput sequencing technologies enable the extraction of vast amounts of genomic data from a single sample, facilitating the utilization of bioinformatics tools to uncover valuable clinical insights. This study identified 126 intersection genes through differential gene analysis and WGCNA analysis on the GSE197884 dataset. GO functional analysis of the intersection genes revealed that they were mainly enriched in fat metabolism, small molecule metabolism, alcohol metabolism, and cell reaction. In addition, KEGG pathway analysis indicated that the genes were mainly associated with retinol metabolism, chemical carcinogens, drug metabolism, amino acid degradation, arachidonic acid metabolism, and PPAR metabolism pathways. These results suggest that the genes are associated with the proliferation and apoptosis of liver cancer cells, the liver's digestion and metabolism, the tumor microenvironment of liver cancer, and the body's immune responses. The identified genes might regulate lipid metabolism and fat formation through MAPK, PPAR, and other signaling pathways, maintain metabolic stability and the expression of inflammatory genes, and play an induction or anti-cancer role in various human tumors.

Furthermore, the MCODE plug-in of Cytoscape identified 11 core genes, among which seven hub genes were discovered after validation using data retrieved from the TCGA database. Among them, *NSDHL* and *SQLE* were upregulated in LIHC tissues, whereas *MVK, GCAT, ALAS2, GLDC*, and *AGXT* were downregulated. This study focused on the role of seven hub genes in HCC induced by HFD/SW. *SQLE*, one of the two rate-limiting enzymes involved in cholesterol synthesis, plays a pivotal role in tumor development and is strongly associated with unfavorable patient outcomes [44-46]. The RNA sequencing analysis revealed that *SQLE* is the predominant metabolic gene overexpressed in patients with NAFLD-HCC. Specific transgenic expression of Sqle in mouse hepatocytes significantly accelerates the progression of HCC induced by a high-fat, high-cholesterol diet. In NAFLD-HCC, augmented *SQLE* expression promotes cholesterol ester biosynthesis, thereby stimulating cellular proliferation [47]. *MVK* serves as a pivotal catalyst in the mevalonate pathway, an intricate biosynthetic route responsible for the production of cholesterol and non-sterol isoprenoids [48]. Insertional activation of *MVK* is caused by hepatitis B virus DNA in a human hepatoma cell line, which phosphorylates mevalonate, a crucial intermediate in the biosynthesis pathway of branched cholesterol/isoprene. Isoprene acylation plays an indispensable role in modulating the function of cellular proteins involved in growth control, including proto-oncogenes [49]. The involvement of *GCAT* in the reprogramming of mitochondrial bioenergetics has been extensively reported in bladder cancer, non-small cell lung cancer, and colon cancer. Furthermore, its association with cancer prognosis highlights its significance; however, a comprehensive understanding of its specific mechanism in NAFLD-HCC necessitates further investigation [50, 51]. According to literature reports, *ALAS2* is a crucial rate-limiting enzyme in the heme synthesis pathway and exhibits a close association with oxidative stress [52, 53]. *ALAS2* and its associated metabolic pathways may potentially exert a pivotal role in the pathogenesis of epirubicin-induced cardiomyopathy among breast cancer patients [54]. However, the potential involvement of *ALAS2* in hepatocellular carcinoma associated with metabolic disorders remains unexplored. *GLDC* is a pivotal enzyme in glycine metabolism, exerting significant influence on cancer glycolytic metabolism, invasion, and metastasis, as well as immune evasion [55]. The previous study demonstrated that elevated levels of *GLDC* expression are correlated with improved overall survival and serve as an independent prognostic factor in patients with HCC. Moreover, overexpression of *GLDC* significantly induces autophagy in HCC cells, whereas downregulation of *GLDC* markedly attenuates autophagic activity in these cells [56]. *GLDC* downregulation promotes HCC progression and intrahepatic metastasis by attenuating cofilin ubiquitination mediated by reactive oxygen species [57]. The liver-specific enzyme *AGXT* plays a pivotal role in the catalysis of glyoxylic acid to glycine formation [58, 59]. In both mouse and human NASH, we observed a significant inhibition of *AGXT1* and identified an inverse correlation between *AGXT1* expression and hepatic lipid accumulation in humans [60]. The *AGXT* downregulation indicated inadequate differentiation of HCC. It serves as a prognostic indicator for unfavorable survival outcomes. Moreover, the knockdown of *AGXT* promotes the proliferation and migration capabilities of HCC cell lines [61].

Importantly, the expression of *NSDHL* may be closely associated with the clinical prognosis of LIHC induced by high-fat and high-sugar diets. The expression of *NSDHL* exhibited a positive correlation with the abundance of B cells and neutrophils while displaying a negative correlation with the expression levels of CD4+ cells and monocytes. The cholesterol synthesis pathway, ubiquitous in animal cells, modulates cholesterol production. *NSDHL*, an enzyme involved in downstream cholesterol biosynthesis, is mainly located in the endoplasmic reticulum membrane and lipid droplets where enzymatic reactions occur [62]. It catalyzes the oxidative decarboxylation of C4 methyl in meiosis-activating sterols and plays a key role in cholesterol synthesis. The loss-of-function mutations of human *NSDHL* genes have been documented to be associated with congenital hemidysplasia with ichthyosiform nevus and limb defects syndrome in previous studies [63, 64]. However, only a few studies have explored the role of *NSDHL* in cancers. For instance, proteomics analysis confirmed that the translocation of *NSDHL* from the intracellular compartment to the plasma membrane might be necessary for cancer metastasis and progression [65]. Previous studies also revealed that malignant tumors, such as breast cancer [66], pancreatic cancer [67], and gastric cancers [68], have higher expression levels of *NSDHL* than adjacent control tissues. The inactivation of *NSDHL* in skin tumors can induce the expression of ATP-binding cassette transporters and reduce low-density lipoprotein receptors and intracellular cholesterols, mainly depending on the liver X receptor. In addition, one study proved that the accumulation of sterol metabolites caused by the lack of *NSDHL* could inhibit tumor growth [69]. Sukhanova *et al.* [70] found that the loss of *NSDHL* expression enhanced the sensitivity of cancer cells to EGFR-targeted inhibitors. Altogether, the findings of this study, combined with the literature mentioned above, suggest that *NSDHL* can serve as a specific biomarker of liver cancer induced by high-fat and high-sugar diets, which provides a basis for precisely targeted therapy in the future.

However, this study also has some limitations. First, there is a shortage of research data because the study was mainly based on data retrieved from online databases. Given that the databases are constantly supplemented and improved with new data, the results described here may be biased. Second, the study has not provided a precise therapy. Third, *in vitro* and *in vivo* experiments and prospective clinical observations should be conducted to validate our results. Finally, potential biases inherent in using specific datasets or limitations related to sample sizes should be acknowledged and discussed.

## CONCLUSION

This study aimed to explore the pathogenesis and potential biomarkers of HCC induced by HFD/SW through bioinformatics strategies. We identified seven hub genes (*NSDHL, MVK, SQLE, GCAT, ALAS2, GLDC*, and *AGXT*) with potential prognostic value, among which *NSDHL* exhibited a significant association with the survival and prognosis of HCC induced by HFD/SW. Our study suggested that *NSDHL* can serve as a specific biomarker for predicting the clinical diagnosis and prognosis of HCC patients, providing a new strategy for targeted therapy and prognosis evaluation of HCC induced by metabolic dysregulation. In the future, we advocate for the establishment of a comprehensive human HCC sample repository to investigate distinct biological markers associated with HCC development from diverse etiologies, thereby facilitating precise clinical interventions.

## AUTHORS’ CONTRIBUTIONS

The authors confirm their contribution to the paper as follows: X.F.Z. and J.H. designed the study. J.H., B.P., and Y.S.L. performed the study and their analyses. B.P. and L.Y.S. were responsible for data collection. Z.X.Y. and X.F.Z. performed data analyses. All authors participated in data interpretation, manuscript review, and writing. B.P. and J.H. were responsible for the preparation of the figures.

All authors reviewed the results and approved the final version of the manuscript.

## Figures and Tables

**Fig. (5):**
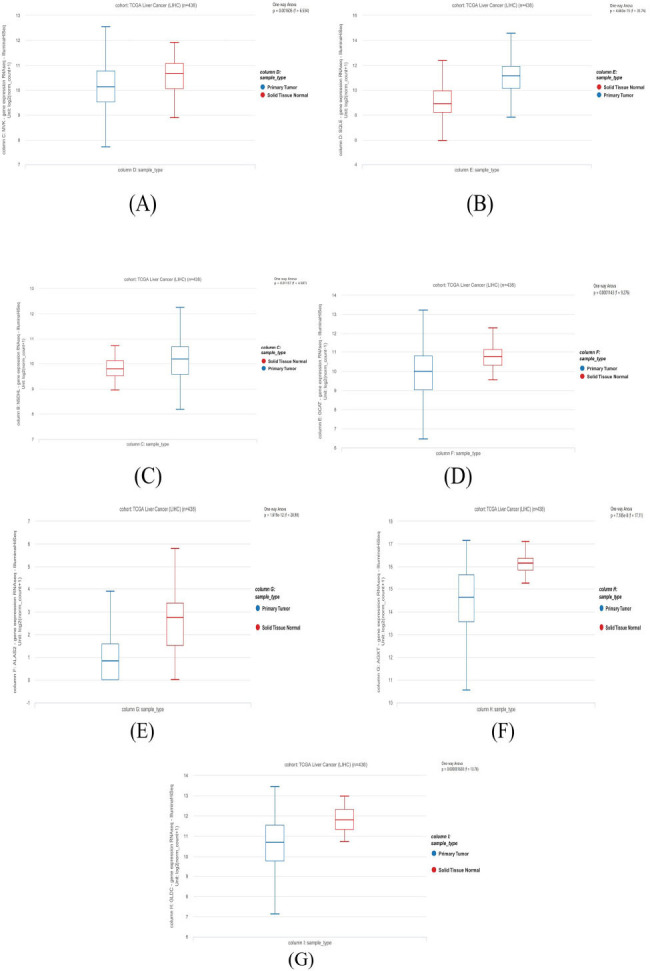
Validation of the expression levels of the ten hub genes in liver hepatocellular carcinoma (LIHC) and normal tissues was performed using data from the TCGA database. (**A**) The gene expression value MVK was assessed among samples from TCGA. (**B**) The gene expression value SQLE was evaluated among samples from TCGA. (**C**) The gene expression value *NSDHL* was examined among samples from TCGA. (**D**) The gene expression value GCAT was analyzed among samples from TCGA. (**E**) The gene expression value ALAS2 was measured among samples from TCGA. (**F**) The gene expression value AGXT was determined among samples from TCGA. (**G**) The gene expression value GLDC was quantified among samples from TCGA.

**Fig. (7):**
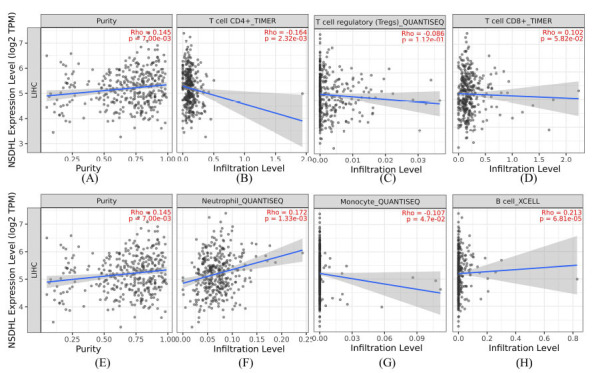
**(A-H)** The correlation between *NSDHL* expression and infiltration levels of CD8+ T cells, CD4+ T cells, Treg cells, B cells, neutrophils, myeloid dendritic cells, and monocytes in LIHC can be found in the TIMER2.0 database.

## Data Availability

The data and supportive information are available within the article.

## LIST OF ABBREVIATIONS

HCC: Hepatocellular Carcinoma
DEGs: Differentially Expressed Genes
WGCNA: Weighted Gene co-expression Analysis
PPI: Protein-Protein Interaction
TCGA: The Cancer Genome Atlas
GO: Gene Ontology
KEGG: Kyoto Encyclopedia of Genes and Genomes
NAFLD: Non-Alcoholic Fatty Liver Disease
NASH: Nonalcoholic Steatohepatitis
GEO: Gene Expression Omnibus
HFD/SW: High-Fat Diet and Sugar Wate
MCODE: Molecular Complex Detection
LIHC: Liver Hepatocellular Carcinoma
